# Poly[aqua­[μ_3_-*N*′-(carboxy­meth­yl)ethyl­ene­diamine-*N*,*N*,*N*′-triacetato]samarium(III)]

**DOI:** 10.1107/S1600536808031036

**Published:** 2008-09-30

**Authors:** Guo-Yong Zhou, Gui-Rong Wu, Zhi-Yong Deng, Xing-Tian Chen

**Affiliations:** a Department of Chemistry and Biology Engineering, Hezhou University, Hezhou, Guangxi Zhuang Autonomous Region 542800, People’s Republic of China

## Abstract

In the title coordination polymer, [Sm(C_10_H_13_N_2_O_8_)(H_2_O)]_*n*_, each samarium(III) centre is nine-coordinated by six O and two N atoms from three *N*′-(carboxy­meth­yl)ethyl­enediamine-*N*,*N*,*N*′-triacetate ligands and one O atom of a water mol­ecule, forming polymeric chains running parallel to the *a* axis. The packing is governed by inter­molecular O—H⋯O hydrogen-bonding inter­actions.

## Related literature

For the corresponding neodymium polymeric complex, see: Huang *et al.* (2008 ▶). For related literature, see: Dakanali *et al.* (2003 ▶); Kitaura *et al.* (2002 ▶); Rowsell *et al.* (2004 ▶).
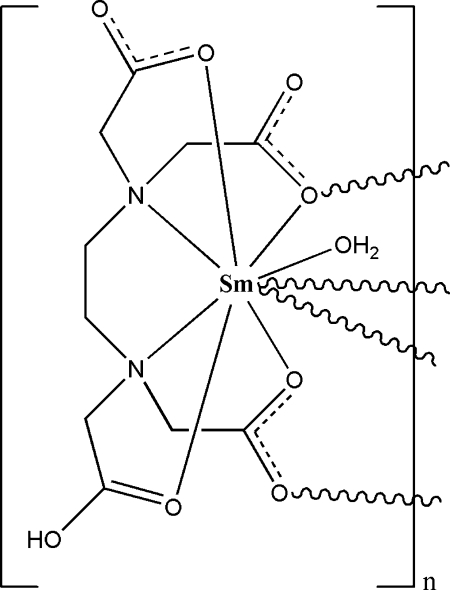


## Experimental

### 

#### Crystal data

[Sm(C_10_H_13_N_2_O_8_)(H_2_O)]
*M* *_r_* = 457.60
Orthorhombic, 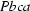

*a* = 6.6506 (7) Å
*b* = 14.7051 (16) Å
*c* = 25.967 (3) Å
*V* = 2539.5 (5) Å^3^
*Z* = 8
Mo *K*α radiation
μ = 4.68 mm^−1^
*T* = 296 (2) K
0.23 × 0.19 × 0.18 mm

#### Data collection

Bruker APEXII area-detector diffractometer
Absorption correction: multi-scan (*SADABS*; Sheldrick, 1996 ▶) *T*_min_ = 0.355, *T*_max_ = 0.433
13066 measured reflections
2637 independent reflections
2289 reflections with *I* > 2σ(*I*)
*R*_int_ = 0.035

#### Refinement

*R*[*F* ^2^ > 2σ(*F* ^2^)] = 0.028
*wR*(*F* ^2^) = 0.064
*S* = 1.03
2637 reflections
206 parameters
3 restraints
H atoms treated by a mixture of independent and constrained refinement
Δρ_max_ = 0.94 e Å^−3^
Δρ_min_ = −1.21 e Å^−3^

### 

Data collection: *APEX2* (Bruker, 2004 ▶); cell refinement: *SAINT* (Bruker, 2004 ▶); data reduction: *SAINT*; program(s) used to solve structure: *SHELXS97* (Sheldrick, 2008 ▶); program(s) used to refine structure: *SHELXL97* (Sheldrick, 2008 ▶); molecular graphics: *SHELXTL* (Sheldrick, 2008 ▶); software used to prepare material for publication: *SHELXTL*.

## Supplementary Material

Crystal structure: contains datablocks I. DOI: 10.1107/S1600536808031036/rz2243sup1.cif

Structure factors: contains datablocks I. DOI: 10.1107/S1600536808031036/rz2243Isup2.hkl

Additional supplementary materials:  crystallographic information; 3D view; checkCIF report

## Figures and Tables

**Table 1:** Hydrogen-bond geometry (Å, °)

*D*—H⋯*A*	*D*—H	H⋯*A*	*D*⋯*A*	*D*—H⋯*A*
O4—H4⋯O2^i^	0.82	1.66	2.474 (4)	170
O1*W*—H1*W*⋯O6^ii^	0.820 (10)	2.02 (2)	2.771 (4)	152.8 (18)
O1*W*—H2*W*⋯O8^iii^	0.823 (10)	2.10 (2)	2.928 (4)	177.1 (15)

Symmetry codes: (i) 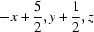; (ii) 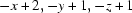; (iii) 
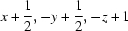
.

**Fig. 1.**
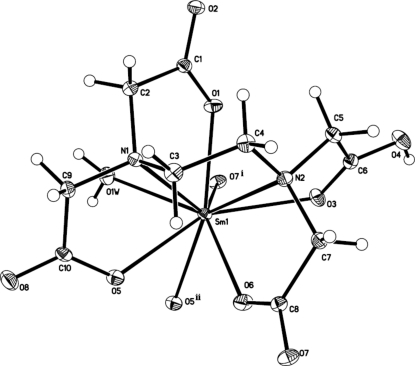
The structure of the title compound, showing the atomic numbering scheme. Non-H atoms are shown as 30% probability displacement ellipsoids. Symmetry codes: (i: 1+x, y, z; ii: 2-x, 1-y, 1-z).

**Fig. 2.**
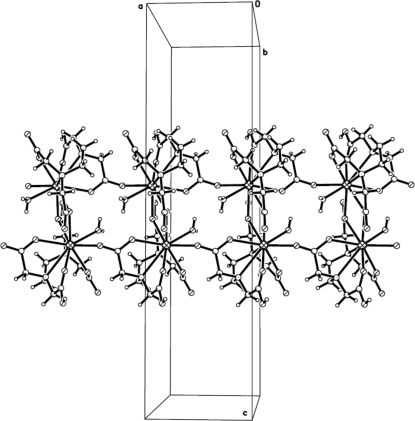
The one-dimensional polymeric chain of the title compound.

## supplementary crystallographic information

### Comment

Research on metal–organic frameworks has been expanding rapidly, due to their interesting structural motifs (Dakanali *et al.*, 2003) and other potential applications (Kitaura *et al.*, 2002; Rowsell *et al.*, 2004) in molecular-based materials. Ethylenediaminetetraacetic acid (H_4_edta) is a good example of a bridging ligand that can link metal centres into extended networks. Herein, we report a new samarium complex obtained by the hydrothermal treatment of Sm_2_O_3_ and H_4_edta in the presence of HClO_4_.

The samarium(III) metal centre is nine-coordinated by six oxygen and two nitrogen atoms from three different N'-(carboxymethyl)ethylenediamine-N,N,N'-triacetato ligands and one water molecule (Fig. 1) to form a polymeric chain running parallel to the crystallographic *a* axis (Fig. 2). The Sm···Sm separations between adjacent metal centres are 4.2461 (6) and 6.6506 (8) Å. The polymeric chains self-assemble *via* intermolecular O—H···O hydrogen bonding interactions (Table 1) to form a three-dimensional supramolecular network. The title compound is isostructural with the corresponding neodymium polymeric complex (Huang *et al.*, 2008).

### Experimental

A mixture of Sm_2_O_3_ (0.5 mmol), ethylenediaminetetraacetic acid (H_4_edta) (0.5 mmol), HClO_4_ (0.2 mmol) and H_2_O (10 ml) was placed in a 23 ml Teflon reactor, which was heated to 433 K for three days and then cooled to room temperature at a rate of 10 K h^-1^. The crystals obtained were washed with water and dryed in air.

### Refinement

Water H atoms were tentatively located in difference Fourier maps and were refined with distance restraints of O—H = 0.82 Å and H···H = 1.20 Å, each within a standard deviation of 0.01 Å, and with *U*_iso_(H) = 1.5*U*_eq_(O). Other H atoms were placed in calculated positions (C—H = 0.97 Å and O—H = 0.82 Å) and refined using a riding model, with *U*_iso_(H) = 1.2*U*_eq_(C, O).

### Crystal data

**Table:**

[Sm(C_10_H_13_N_2_O_8_)(H_2_O)]	*F*(000) = 1784
*M**_r_* = 457.60	*D*_x_ = 2.394 Mg m^−^^3^
Orthorhombic, *P**b**c**a*	Mo *K*α radiation, λ = 0.71073 Å
Hall symbol: -P 2ac 2ab	Cell parameters from 3600 reflections
*a* = 6.6506 (7) Å	θ = 1.7–28.0°
*b* = 14.7051 (16) Å	µ = 4.68 mm^−^^1^
*c* = 25.967 (3) Å	*T* = 296 K
*V* = 2539.5 (5) Å^3^	Block, colourless
*Z* = 8	0.23 × 0.19 × 0.18 mm

### Data collection

**Table:**

Bruker APEXII area-detector diffractometer	2637 independent reflections
Radiation source: fine-focus sealed tube	2289 reflections with *I* > 2σ(*I*)
graphite	*R*_int_ = 0.035
φ and ω scan	θ_max_ = 26.5°, θ_min_ = 1.6°
Absorption correction: multi-scan (SADABS; Sheldrick, 1996)	*h* = −8→5
*T*_min_ = 0.355, *T*_max_ = 0.433	*k* = −16→18
13066 measured reflections	*l* = −32→32

### Refinement

**Table:**

Refinement on *F*^2^	Primary atom site location: structure-invariant direct methods
Least-squares matrix: full	Secondary atom site location: difference Fourier map
*R*[*F*^2^ > 2σ(*F*^2^)] = 0.028	Hydrogen site location: inferred from neighbouring sites
*wR*(*F*^2^) = 0.064	H atoms treated by a mixture of independent and constrained refinement
*S* = 1.03	*w* = 1/[σ^2^(*F*_o_^2^) + (0.0288*P*)^2^ + 6.2721*P*] where *P* = (*F*_o_^2^ + 2*F*_c_^2^)/3
2637 reflections	(Δ/σ)_max_ = 0.002
206 parameters	Δρ_max_ = 0.94 e Å^−^^3^
3 restraints	Δρ_min_ = −1.21 e Å^−^^3^

### Special details

**Table:**

Geometry. All e.s.d.'s (except the e.s.d. in the dihedral angle between two l.s. planes) are estimated using the full covariance matrix. The cell e.s.d.'s are taken into account individually in the estimation of e.s.d.'s in distances, angles and torsion angles; correlations between e.s.d.'s in cell parameters are only used when they are defined by crystal symmetry. An approximate (isotropic) treatment of cell e.s.d.'s is used for estimating e.s.d.'s involving l.s. planes.
Refinement. Refinement of *F*^2^ against ALL reflections. The weighted *R*-factor *wR* and goodness of fit *S* are based on *F*^2^, conventional *R*-factors *R* are based on *F*, with *F* set to zero for negative *F*^2^. The threshold expression of *F*^2^ > σ(*F*^2^) is used only for calculating *R*-factors(gt) *etc*. and is not relevant to the choice of reflections for refinement. *R*-factors based on *F*^2^ are statistically about twice as large as those based on *F*, and *R*- factors based on ALL data will be even larger.

### Fractional atomic coordinates and isotropic or equivalent isotropic displacement parameters (Å^2^)

**Table:**

	*x*	*y*	*z*	*U*_iso_*/*U*_eq_	
Sm1	1.07590 (3)	0.506856 (12)	0.420681 (7)	0.01387 (8)	
C1	1.2133 (6)	0.3523 (3)	0.33453 (14)	0.0168 (8)	
C2	1.0904 (6)	0.2870 (3)	0.36651 (15)	0.0186 (8)	
H2A	1.0170	0.2467	0.3437	0.022*	
H2B	1.1807	0.2500	0.3870	0.022*	
C3	0.7461 (6)	0.3440 (3)	0.37554 (15)	0.0197 (8)	
H3A	0.6543	0.3739	0.3991	0.024*	
H3B	0.6918	0.2843	0.3678	0.024*	
C4	0.7586 (6)	0.3985 (3)	0.32648 (14)	0.0193 (8)	
H4A	0.8580	0.3713	0.3039	0.023*	
H4B	0.6297	0.3965	0.3090	0.023*	
C5	0.9165 (6)	0.5334 (3)	0.29164 (15)	0.0186 (8)	
H5A	0.8189	0.5486	0.2652	0.022*	
H5B	1.0097	0.4893	0.2774	0.022*	
C6	1.0293 (6)	0.6179 (3)	0.30788 (15)	0.0178 (8)	
C7	0.6310 (6)	0.5491 (3)	0.34790 (15)	0.0199 (9)	
H7A	0.5163	0.5205	0.3315	0.024*	
H7B	0.6467	0.6091	0.3329	0.024*	
C8	0.5880 (6)	0.5592 (3)	0.40475 (15)	0.0166 (8)	
C9	0.9173 (6)	0.2796 (3)	0.44879 (15)	0.0194 (9)	
H9A	1.0277	0.2369	0.4522	0.023*	
H9B	0.7942	0.2447	0.4458	0.023*	
C10	0.9064 (6)	0.3372 (3)	0.49695 (15)	0.0173 (8)	
N1	0.9459 (5)	0.3332 (2)	0.40114 (12)	0.0164 (7)	
N2	0.8138 (5)	0.4946 (2)	0.33643 (12)	0.0173 (7)	
O1	1.2293 (4)	0.43367 (18)	0.34962 (10)	0.0207 (6)	
O2	1.2980 (5)	0.32419 (18)	0.29435 (10)	0.0235 (7)	
O3	1.0724 (4)	0.63138 (19)	0.35307 (11)	0.0232 (7)	
O4	1.0758 (5)	0.6717 (2)	0.27029 (11)	0.0292 (7)	
H4	1.1182	0.7199	0.2819	0.044*	
O5	0.9074 (4)	0.42346 (18)	0.49227 (10)	0.0203 (6)	
O6	0.7369 (4)	0.5605 (2)	0.43483 (10)	0.0233 (6)	
O7	0.4107 (4)	0.57144 (19)	0.41872 (11)	0.0211 (6)	
O8	0.9000 (5)	0.2985 (2)	0.53938 (11)	0.0294 (7)	
O1W	1.3250 (5)	0.3977 (2)	0.46232 (11)	0.0271 (7)	
H2W	1.350 (8)	0.3428 (10)	0.4613 (15)	0.041*	
H1W	1.348 (8)	0.412 (3)	0.4922 (7)	0.041*	

### Atomic displacement parameters (Å^2^)

**Table:**

	*U*^11^	*U*^22^	*U*^33^	*U*^12^	*U*^13^	*U*^23^
Sm1	0.01036 (12)	0.01782 (12)	0.01343 (12)	0.00028 (8)	0.00067 (7)	−0.00100 (8)
C1	0.0145 (19)	0.021 (2)	0.0153 (18)	0.0028 (16)	0.0001 (15)	−0.0005 (15)
C2	0.019 (2)	0.0179 (19)	0.019 (2)	0.0032 (17)	0.0014 (16)	0.0000 (16)
C3	0.016 (2)	0.0196 (19)	0.023 (2)	−0.0039 (17)	−0.0008 (18)	−0.0007 (16)
C4	0.017 (2)	0.022 (2)	0.0197 (19)	−0.0027 (16)	−0.0040 (17)	−0.0026 (15)
C5	0.019 (2)	0.022 (2)	0.0152 (19)	−0.0017 (17)	−0.0002 (16)	−0.0008 (16)
C6	0.0142 (19)	0.018 (2)	0.021 (2)	0.0000 (16)	0.0007 (16)	0.0010 (16)
C7	0.016 (2)	0.026 (2)	0.018 (2)	0.0035 (17)	0.0008 (16)	0.0034 (17)
C8	0.014 (2)	0.0157 (18)	0.020 (2)	−0.0015 (15)	0.0019 (16)	−0.0007 (15)
C9	0.025 (2)	0.017 (2)	0.016 (2)	0.0001 (17)	0.0005 (17)	0.0022 (15)
C10	0.0102 (19)	0.024 (2)	0.0176 (19)	−0.0010 (16)	0.0031 (15)	0.0005 (16)
N1	0.0181 (18)	0.0169 (16)	0.0143 (16)	0.0004 (14)	0.0019 (13)	0.0000 (13)
N2	0.0138 (16)	0.0193 (17)	0.0187 (16)	0.0011 (14)	0.0008 (13)	0.0005 (14)
O1	0.0174 (15)	0.0206 (14)	0.0242 (14)	−0.0011 (12)	0.0052 (12)	−0.0055 (12)
O2	0.0319 (18)	0.0201 (14)	0.0186 (14)	0.0006 (13)	0.0078 (12)	−0.0020 (12)
O3	0.0277 (17)	0.0257 (16)	0.0163 (14)	−0.0039 (13)	−0.0037 (12)	0.0008 (12)
O4	0.046 (2)	0.0227 (16)	0.0190 (15)	−0.0149 (15)	0.0017 (14)	0.0007 (12)
O5	0.0218 (16)	0.0184 (14)	0.0209 (15)	−0.0051 (12)	0.0034 (12)	−0.0039 (11)
O6	0.0133 (14)	0.0355 (17)	0.0211 (14)	0.0051 (13)	−0.0039 (12)	−0.0038 (12)
O7	0.0115 (14)	0.0224 (15)	0.0295 (16)	−0.0004 (11)	0.0016 (12)	−0.0047 (12)
O8	0.0337 (19)	0.0355 (18)	0.0189 (15)	0.0007 (15)	0.0027 (13)	0.0061 (13)
O1W	0.0303 (18)	0.0290 (17)	0.0221 (16)	0.0045 (15)	−0.0043 (14)	0.0029 (13)

### Geometric parameters (Å, °)

**Table:**

Sm1—O1	2.367 (3)	C5—C6	1.512 (5)
Sm1—O6	2.416 (3)	C5—H5A	0.9700
Sm1—O7^i^	2.421 (3)	C5—H5B	0.9700
Sm1—O5^ii^	2.484 (3)	C6—O3	1.224 (5)
Sm1—O5	2.493 (3)	C6—O4	1.294 (5)
Sm1—O3	2.537 (3)	C7—N2	1.486 (5)
Sm1—O1W	2.548 (3)	C7—C8	1.511 (5)
Sm1—N1	2.744 (3)	C7—H7A	0.9700
Sm1—N2	2.803 (3)	C7—H7B	0.9700
C1—O2	1.256 (4)	C8—O7	1.247 (5)
C1—O1	1.263 (5)	C8—O6	1.261 (5)
C1—C2	1.510 (5)	C9—N1	1.479 (5)
C2—N1	1.481 (5)	C9—C10	1.512 (5)
C2—H2A	0.9700	C9—H9A	0.9700
C2—H2B	0.9700	C9—H9B	0.9700
C3—N1	1.494 (5)	C10—O8	1.241 (5)
C3—C4	1.507 (5)	C10—O5	1.274 (5)
C3—H3A	0.9700	O4—H4	0.8200
C3—H3B	0.9700	O5—Sm1^ii^	2.484 (3)
C4—N2	1.483 (5)	O7—Sm1^iii^	2.421 (3)
C4—H4A	0.9700	O1W—H2W	0.823 (10)
C4—H4B	0.9700	O1W—H1W	0.820 (10)
C5—N2	1.464 (5)		
			
O1—Sm1—O6	131.98 (9)	N2—C4—H4B	109.2
O1—Sm1—O7^i^	76.45 (9)	C3—C4—H4B	109.2
O6—Sm1—O7^i^	137.16 (10)	H4A—C4—H4B	107.9
O1—Sm1—O5^ii^	151.34 (10)	N2—C5—C6	109.3 (3)
O6—Sm1—O5^ii^	76.64 (9)	N2—C5—H5A	109.8
O7^i^—Sm1—O5^ii^	79.40 (9)	C6—C5—H5A	109.8
O1—Sm1—O5	123.46 (9)	N2—C5—H5B	109.8
O6—Sm1—O5	68.14 (9)	C6—C5—H5B	109.8
O7^i^—Sm1—O5	128.46 (9)	H5A—C5—H5B	108.3
O5^ii^—Sm1—O5	62.91 (10)	O3—C6—O4	124.6 (4)
O1—Sm1—O3	78.03 (9)	O3—C6—C5	121.1 (4)
O6—Sm1—O3	82.03 (9)	O4—C6—C5	114.2 (3)
O7^i^—Sm1—O3	73.18 (9)	N2—C7—C8	113.8 (3)
O5^ii^—Sm1—O3	109.41 (9)	N2—C7—H7A	108.8
O5—Sm1—O3	150.11 (9)	C8—C7—H7A	108.8
O1—Sm1—O1W	76.36 (9)	N2—C7—H7B	108.8
O6—Sm1—O1W	138.39 (10)	C8—C7—H7B	108.8
O7^i^—Sm1—O1W	70.02 (10)	H7A—C7—H7B	107.7
O5^ii^—Sm1—O1W	81.07 (10)	O7—C8—O6	124.1 (4)
O5—Sm1—O1W	70.48 (10)	O7—C8—C7	118.5 (3)
O3—Sm1—O1W	138.98 (10)	O6—C8—C7	117.2 (3)
O1—Sm1—N1	64.43 (9)	N1—C9—C10	113.6 (3)
O6—Sm1—N1	92.16 (10)	N1—C9—H9A	108.9
O7^i^—Sm1—N1	130.62 (9)	C10—C9—H9A	108.9
O5^ii^—Sm1—N1	124.49 (9)	N1—C9—H9B	108.9
O5—Sm1—N1	62.50 (9)	C10—C9—H9B	108.9
O3—Sm1—N1	122.76 (9)	H9A—C9—H9B	107.7
O1W—Sm1—N1	72.34 (10)	O8—C10—O5	122.8 (4)
O1—Sm1—N2	68.30 (10)	O8—C10—C9	118.6 (4)
O6—Sm1—N2	63.86 (9)	O5—C10—C9	118.6 (3)
O7^i^—Sm1—N2	125.50 (9)	C9—N1—C2	110.3 (3)
O5^ii^—Sm1—N2	139.87 (9)	C9—N1—C3	108.3 (3)
O5—Sm1—N2	105.71 (9)	C2—N1—C3	110.8 (3)
O3—Sm1—N2	60.04 (9)	C9—N1—Sm1	112.4 (2)
O1W—Sm1—N2	134.02 (10)	C2—N1—Sm1	109.5 (2)
N1—Sm1—N2	66.42 (9)	C3—N1—Sm1	105.3 (2)
O2—C1—O1	122.1 (4)	C5—N2—C4	110.4 (3)
O2—C1—C2	119.3 (3)	C5—N2—C7	109.3 (3)
O1—C1—C2	118.5 (3)	C4—N2—C7	110.2 (3)
N1—C2—C1	113.2 (3)	C5—N2—Sm1	107.8 (2)
N1—C2—H2A	108.9	C4—N2—Sm1	110.6 (2)
C1—C2—H2A	108.9	C7—N2—Sm1	108.5 (2)
N1—C2—H2B	108.9	C1—O1—Sm1	129.5 (2)
C1—C2—H2B	108.9	C6—O3—Sm1	123.3 (3)
H2A—C2—H2B	107.8	C6—O4—H4	109.5
N1—C3—C4	112.6 (3)	C10—O5—Sm1^ii^	108.9 (2)
N1—C3—H3A	109.1	C10—O5—Sm1	124.3 (2)
C4—C3—H3A	109.1	Sm1^ii^—O5—Sm1	117.09 (10)
N1—C3—H3B	109.1	C8—O6—Sm1	129.3 (2)
C4—C3—H3B	109.1	C8—O7—Sm1^iii^	145.0 (3)
H3A—C3—H3B	107.8	Sm1—O1W—H2W	137 (3)
N2—C4—C3	111.9 (3)	Sm1—O1W—H1W	111 (3)
N2—C4—H4A	109.2	H2W—O1W—H1W	104 (4)
C3—C4—H4A	109.2		

Symmetry codes: (i) *x*+1, *y*, *z*; (ii) −*x*+2, −*y*+1, −*z*+1; (iii) *x*−1, *y*, *z*.

### Hydrogen-bond geometry (Å, °)

**Table:**

*D*—H···*A*	*D*—H	H···*A*	*D*···*A*	*D*—H···*A*
O4—H4···O2^iv^	0.82	1.66	2.474 (4)	170.
O1W—H1W···O6^ii^	0.82 (1)	2.02 (2)	2.771 (4)	153.(2)
O1W—H2W···O8^v^	0.82 (1)	2.10 (2)	2.928 (4)	177.(2)

Symmetry codes: (iv) −*x*+5/2, *y*+1/2, *z*; (ii) −*x*+2, −*y*+1, −*z*+1; (v) *x*+1/2, −*y*+1/2, −*z*+1.
